# *Ligilactobacillus animalis* 506 Protects the Intestinal Barrier from the Damaging Effects of Enteric Pathogens and Deoxynivalenol

**DOI:** 10.3390/ani14020269

**Published:** 2024-01-15

**Authors:** Erik Juncker Boll, Katrine Damgaard Winther, Tine Thorup Møller Knudsen, Giuseppe Copani, Bruno Ieda Cappellozza

**Affiliations:** Chr. Hansen, Animal and Plant Health & Nutrition, Boege Allé 10-12, 2970 Hoersholm, Denmarkdkgico@chr-hansen.com (G.C.); brbrie@chr-hansen.com (B.I.C.)

**Keywords:** probiotics, enteric pathogens, gut barrier integrity, inflammation

## Abstract

**Simple Summary:**

The intestinal epithelial barrier is a critical host defense mechanism and plays an important role in the gastrointestinal health of animals. Impaired gut barrier integrity caused by enteric pathogenic infections or ingested mycotoxins is a particular concern in farm animals, as they may trigger intestinal inflammation and possibly chronic pathological conditions. *Ligilactobacillus animalis* (formerly known as *Lactobacillus animalis*) 506 is a probiotic lactic acid-producing bacterial strain used in commercial products to support the health and performance of beef and dairy cattle. However, limited information is available regarding the mechanism underlying its proposed in vivo benefits. This study investigated the ability of *L. animalis* 506 to support gut barrier integrity and inflammation in vitro. The results showed that *L. animalis* 506 supported gut barrier integrity and regulated the release of interleukin (IL)-8 from epithelial cells upon challenge with enteric pathogenic isolates. Moreover, *L. animalis* 506 mitigated epithelial barrier disruption and tight junction protein redistribution caused by the frequent food contaminant mycotoxin deoxynivalenol (DON). These results provide new insights into the potential mechanisms by which *L. animalis* 506 might benefit farm animals.

**Abstract:**

This study investigated the impact of *L. animalis* 506 on gut barrier integrity and regulation of inflammation in vitro using intestinal epithelial cell lines. Caco-2 or HT29 cell monolayers were challenged with enterotoxigenic *E. coli* (ETEC) or a ruminant isolate of *Salmonella* Heidelberg in the presence or absence of one of six probiotic *Lactobacillus* spp. strains. Among these, *L. animalis* 506 excelled at exerting protective effects by significantly mitigating the decreased transepithelial electrical resistance (TEER) as assessed using area under the curve (AUC) (*p* < 0.0001) and increased apical-to-basolateral fluorescein isothiocyanate (FITC) dextran translocation (*p* < 0.0001) across Caco-2 cell monolayers caused by *S*. Heidelberg or ETEC, respectively. Similarly, *L. animalis* 506 and other probiotic strains significantly attenuated the *S*. Heidelberg- and ETEC-induced increase in IL-8 from HT29 cells (*p* < 0.0001). Moreover, *L. animalis* 506 significantly counteracted the TEER decrease (*p* < 0.0001) and FITC dextran translocation (*p* < 0.0001) upon challenge with *Clostridium perfringens*. Finally, *L. animalis* 506 significantly attenuated DON-induced TEER decrease (*p* < 0.01) and FITC dextran translocation (*p* < 0.05) and mitigated occludin and zona occludens (ZO)-1 redistribution in Caco-2 cells caused by the mycotoxin. Collectively, these results demonstrate the ability of *L. animalis* 506 to confer protective effects on the intestinal epithelium in vitro upon challenge with enteric pathogens and DON known to be of particular concern in farm animals.

## 1. Introduction

The intestinal mucosal barrier is a selectively permeable structure that allows the absorption of nutrients while preventing the entry of potentially harmful microorganisms or dietary antigens. A central component of its structure is the intestinal epithelium, which comprises a single layer of cells held closely together by tight junctions. Tight junctions consist of transmembrane proteins, including occludin and claudins, and peripheral membrane proteins, such as zona occludens (ZO)-1, which crosslink the transmembrane proteins and connect tight junctions to the actin cytoskeleton [1]. 

Impairment of the intestinal barrier integrity results in the development of “leaky gut”, which triggers local inflammatory responses and possibly chronic pathological conditions [2,3]. Gastrointestinal infections with pathogenic bacterial species represent a major risk of disrupted gut barrier integrity and function. They are a particular concern in large-scale farm settings, where production animals might be exposed to social and/or environmental stressful conditions, which may make them more susceptible to infections [4]. For instance, enterotoxigenic *Escherichia coli* (ETEC) is a frequent cause of neonatal diarrhea in calves [5], whereas enteric, septicemic, and reproductive diseases caused by *Salmonella* species are highly problematic in dairy cattle [6]. Clostridial infections are another major concern as a cause of necrotic enteritis in poultry and calves [7,8]. 

In addition, certain mycotoxins, particularly trichothecenes such as deoxynivalenol (DON), are increasingly being recognized for their ability to directly impair intestinal barrier integrity and trigger proinflammatory and immunotoxic effects [9]. This mycotoxin is produced by *Fusarium* fungi known to frequently infect various grains in the field or during storage and is one of the most common mycotoxin contaminants in cereal-based food and animal feed [10,11,12,13]. This fact, along with the stability of DON through food processing and its known cytotoxic effects, has made it a major public health concern as a food contaminant for humans and livestock [10,14,15,16,17,18]. DON exerts both acute and chronic toxic effects, with symptoms including diarrhea, vomiting, abdominal pain, and weight loss. At high dosages, DON ingestion may lead to tissue damage and, ultimately, death, whereas chronic exposure to low dosages causes reduced weight gain and altered nutritional efficiency [19]. 

Several studies have shown that strains of probiotic species, including Lactobacilli, can have beneficial effects on gastrointestinal health, including inhibiting pathogenic microorganisms, favoring healthy microbiota compositions, strengthening the intestinal barrier, and regulating host inflammatory responses [20,21,22,23]. *Ligilactobacillus* (former *Lactobacillus*) *animalis* strain 506 is a lactic acid-producing bacteria used in commercial products to support the health and performance of beef and dairy cattle [24,25,26]. However, only limited information is available regarding the mechanisms underlying its proposed in vivo benefits. Here, we characterize in vitro the beneficial effects of *L. animalis* 506, demonstrating its potential for supporting gut barrier integrity and regulating inflammation upon challenge with various enteric pathogens as well as DON.

## 2. Materials and Methods

### 2.1. Bacterial Strains and Growth Conditions

The following six probiotic strains were used in this study: *Ligilactobacillus* (former *Lactobacillus*) *animalis* 506, *Lacticaseibacillus* (former *Lactobacillus*) *rhamnosus* 994, *Lactobacillus acidophilus* 777, *Lactocaseibacillus* (former *Lactobacillus*) *paracasei* subsp. *paracasei* 136, *Limosilactobacillus* (former *Lactobacillus*) *reuteri* 435, and *Lactiplantibacillus* (former *Lactobacillus*) *plantarum* subsp. *plantarum* 432. The following five pathogenic strains were also included: a porcine isolate of enterotoxigenic *Escherichia coli* (ETEC) O138 F18 (9910297-2^STM^, kindly provided by Professor Zentek, Institute of Animal Nutrition, Frei University, Berlin); ruminant isolates of *Salmonella enterica* serovar Heidelberg, *S*. Newport, and *S*. Dublin; and *Clostridium perfringens* type A strain ATCC 13124. 

The probiotic strains were cultured non-agitated and anaerobically overnight at 37 °C in De Man, Rogosa, and Sharpe (MRS, Chr. Hansen, Hoersholm, Denmark) broth. Ten-fold dilution series were prepared from the overnight cultures and incubated under the same conditions as described above. For each strain, late exponential/early stationary phase, reached after overnight growth, was selected based on measures of optical density at 600 nm (OD_600_).

The ETEC strain was grown agitated overnight in Luria Bertani (LB, Chr. Hansen) broth at 37 °C, thus reaching the stationary phase. As described by McCormick et al. [24], the *Salmonella enterica* strains were grown agitated in LB broth at 37 °C for 6 h and then diluted to a 1:1000 ratio in LB and grown non-agitated overnight, thus reaching a late-exponential phase [27]. The *C. perfringens* strain was grown non-agitated in brain heart infusion (BHI, Chr. Hansen) broth at 37 °C overnight, thus reaching the stationary phase.

### 2.2. Cell Culture

The human cancer-derived epithelial intestinal cell lines Caco-2 (ACC 169, DSMZ, passages 5 to 20) and HT29 (ACC 299, DSMZ, Braunschweig, Germany, passages 5 to 20) were maintained in Dulbecco’s Modified Eagle Medium (DMEM, Thermo Fisher Scientific, Waltham, MA, USA) supplemented with non-essential amino acids (Biowest, Nuaillé, France), penicillin-streptomycin-Amphotericin B Solution (Biological Industries, Kibbutz, Israel), and 10% Fetal Bovine Serum (Thermo Fisher Scientific) at 37 °C and 5% CO_2_. 

For the transepithelial electrical resistance assay, Caco-2 cells were seeded on 1.12-cm^2^ transwells (0.4 µm pore size, Corning, NY, USA) at 5 × 10^4^ cells/insert as previously described [28]. The culture medium was changed every 3–4 days. The cells were utilized after 20–22 days, by which time they had reached a confluent, polarized, and differentiated state. 

For the cytokine release assay, HT29 cells were seeded in 24-well plates (Corning, 1.9-cm^2^ pr. well) pre-coated with fibronectin (Sigma-Aldrich, St. Louis, MO, USA) at 1.5 × 10^5^ cells/well as previously described [29]. The cells were utilized after 5 days upon reaching a near-confluent state. 

### 2.3. Transepithelial Electrical Resistance (TEER) and Fluorescein Isothiocyanate (FITC) Dextran Translocation Assays 

Caco-2 cell monolayers were equilibrated overnight in an antibiotic-free cell culture medium in a CellZscope2 system (NanoAnalytics, Münster, Germany). On the day of the experiment, bacterial strains grown to the late-exponential phase (*Lactobacillus* spp. strains and *Salmonella* strains) or stationary phase (ETEC and *C. perfringens*) were washed twice using Hanks balanced salt solution (HBSS, Thermo Fisher Scientific) and resuspended in antibiotic-free cell culture media. The OD_600nm_-normalized bacteria were then added to the apical compartments of the cell monolayers at a concentration of approximately 1 × 10^8^ and 1–2 × 10^7^ CFU/transwell for probiotic and pathogenic strains, respectively. For the mycotoxin experiments, Deoxynivalenol Ready Made Solution (Sigma-Aldrich) was added to the apical and/or basolateral compartments of the cell monolayers to a final concentration of 25–100 µM. The total volumes in the apical and basolateral compartments were 750 µL and 1650 µL, respectively. Hourly measurements of TEER were carried out for up to 20 h post-challenge administration, depending on the experiment. Results are expressed as relative to baseline values before the addition of the probiotic, pathogenic bacteria, and/or DON to the cell monolayers. The area under the curve (AUC) was calculated from curves of relative TEER plotted against time, using the lowest observed value of the negative control curves as a baseline.

For some experiments, FITC dextran-20 kDa (FD20, Sigma-Aldrich), suspended in antibiotic-free culture medium, was added to the apical compartments in a final amount of 400 µg per insert at the start of the experiment. At the end of the experiment, the amount of FD20 translocated to the basolateral compartment was quantified by measuring fluorescence (490 nm emission/520 nm excitation) using a Biotek Synergy H1 microplate reader (Agilent Technologies, Santa Clara, CA, USA). From this, the percentage of translocated FD20 relative to the apically added baseline level was quantified. All assays were performed in at least triplicates and repeated twice with similar outcomes, unless stated otherwise. 

For experiments with dead cells of *L. animalis* 506, these were generated as described by Rhayat et al. [30]. Briefly, late-exponential phase *L. animalis* 506 was OD_600nm_-normalized in antibiotic-free cell culture as described above, after which the bacteria were exposed to 4% formaldehyde for 10 min, washed three times with HBSS, and finally resuspended in antibiotic-free cell culture media to reach a bacterial density equivalent to that of the live bacteria in the assay. The killing of *L. animalis* 506 was confirmed by the absence of colonies on MRS plates.

### 2.4. Immunofluorescence Microscopy

Caco-2 cells seeded on 1.12-cm^2^ transwells were challenged or not with DON (added both apically and basolaterally) in the presence or absence of *L. animalis* 506, as described above. The cells were then submerged in 4% formaldehyde for 10 min, followed by washing. The filters were then cut from the transwells and submerged in blocking buffer (PBS with 2% bovine serum albumin (BSA, Sigma-Aldrich)) for 1 h, washed, and stained for 1 h with rabbit IgG ZO-1 (Thermo Fisher Scientific) and mouse IgG occludin (Santa Cruz Biotechnology, Dallas, TX, USA) diluted at a 1:200 and 1:100 ratio, respectively, in blocking buffer. The filters were then washed thrice in PBS with 0.25% BSA and 0.1% Triton X-100 (Sigma-Aldrich) and stained for 1 h in a dark chamber with goat anti-mouse IgG Alexa Flour 488 (Life Technologies, Erie, UK) and donkey anti-rabbit IgG Alexa Flour 555 (Thermo Fisher Scientific). The filters were then washed twice with PBS and incubated for 5 min in a dark chamber with 300 nM 3′,6′-diamidino-2-phenylindole (DAPI, Thermo Fisher Scientific) in PBS. After final washing thrice with water, coverslips were mounted using a mounting medium (Agilent Technologies), and immunofluorescent images were captured using an EVOS M7000 Imaging system (Thermo Fisher Scientific). The images chosen are representative of the entire micrograph data sets based on two independent experiments with technical duplicates.

### 2.5. IL-8 Release Assay

On the day of the experiment, HT29 cell monolayers were gently washed using HBSS, after which 500 µL of *Lactobacillus* spp. grown and OD_600nm_-normalized in antibiotic-free cell culture media as described above to approximately 2 × 10^8^ CFU/mL were added to the wells (final concentration of 1 × 10^8^ CFU/well). After 2 h, 50 µL of ETEC or *S*. Heidelberg grown and OD_600nm_-normalized in antibiotic-free cell culture media as described above to approximately 1 × 10^6^ and 5 × 10^6^ CFU/mL, respectively, were added to the wells (final concentrations of approximately 1 × 10^5^ and 5 × 10^5^ CFU/well, respectively). After an additional 3 h of *Lactobacillus* spp./pathogen co-incubation, the cell monolayers were gently washed twice using HBSS, after which fresh cell culture medium containing 50 µg/mL gentamycin (Sigma-Aldrich) was added to the wells. After an additional 20 h, the cell supernatants were collected, and their interleukin (IL)-8 contents were analyzed using a Human IL-8 ELISA kit (Thermo Fisher Scientific) as pr. manufacturer’s instructions.

### 2.6. Statistical Analysis

Data were analyzed in an individual experiment and not between experiments because of variations in TEER between monolayers (baseline resistance, 300–350 Ω × cm^2^). However, the overall trends within an experiment were reproducible between experiments. All TEER, AUC, and FD20 translocation results are expressed as the mean + standard deviation (SD) of an individual experiment performed in triplicate and repeated at least twice, unless stated otherwise. All IL-8 release results are expressed as means + standard error of the mean (SEM) of two combined individual experiments performed in quadruplicate. Differences between the respective “challenge only” treatment groups were conducted using a one-way ANOVA test followed by Dunnett’s multiple comparison tests. *p*-values below 0.05 were considered statistically significant.

## 3. Results

### 3.1. L. animalis 506 Excels among Probiotic Lactobacillus *spp.* Strains at Mitigating Salmonella- and ETEC-Induced Impaired Gut Barrier Integrity 

We first assessed the ability of *L. animalis* 506 and five additional *Lactobacillus* spp. strains to counteract the intestinal barrier damage caused by a strain of *Salmonella enterica* serovar Heidelberg and ETEC, respectively. We measured TEER and apical-to-basolateral translocation of FD20 across differentiated and polarized monolayers of Caco-2 cells, which are well known to acquire structural and functional characteristics of small-intestinal villus cells when grown under these conditions [31]. The *Lactobacillus* spp. strains and ETEC were administered at 1 × 10^8^ and 1 × 10^7^ CFU/transwell, respectively, as previously described [28]. *Salmonella enterica* Heidelberg was administered at 2 × 10^7^ CFU/transwell, based on a preliminary dose–response study.

**Figure 1:**
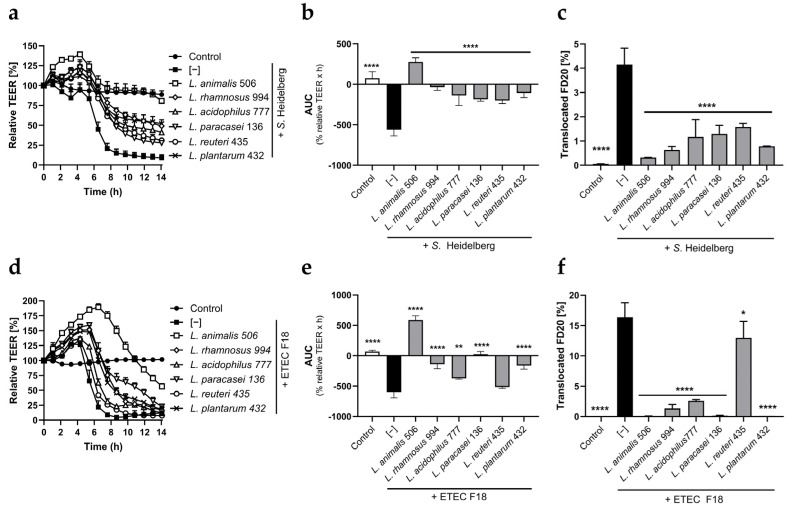
*L. animalis* 506 excels among the probiotic strains at mitigating *Salmonella*- and ETEC-induced impaired gut barrier integrity. Caco-2 cell monolayers were exposed on the apical side to *S*. Heidelberg (**a**–**c**) or ETEC F18 (**d**–**f**) in the presence or absence of probiotic *Lactobacillus* spp. strains and in the presence of FD20. The TEER was measured for a total of 14 h, after which the amount of FD20 translocated to the basolateral compartment was quantified. Data are expressed as means of relative TEER (**a**,**d**), normalized AUC (**b**,**e**), and translocated FD20 (**c**,**f**) + SD (n = 3). **** (*p* < 0.0001), ** (*p* < 0.01), and * (*p* < 0.05) indicate significant differences from the “pathogen only” groups.

The ETEC initially caused a slight TEER increase, which may reflect initial stress on the Caco-2 cells that caused their tight junctions and adherence junctions to interact more tightly. After 4 h, both *S*. Heidelberg and ETEC started to profoundly impair the integrity of the Caco-2 monolayers, with an approximately 80% decrease in TEER observed after 7–8 h (Figure 1a,d). Correspondingly, both pathogens caused a significant increase in FD20 translocation quantified at the end of the 14 h experiments compared to the unstimulated cells (Figure 1c,f). All six probiotic strains were able to significantly alleviate *S*. Heidelberg-induced TEER decrease and FD20 translocation, with *L. animalis* 506 exhibiting the highest level of counteracting effect (Figure 1a–c). The same overall pattern was observed upon ETEC challenge, with *L. animalis* 506 again demonstrating the strongest protective effect (Figure 1d–f). 

We further assessed the potential protective effect of formaldehyde-killed *L. animalis* 506 in the ETEC and *S*. Heidelberg TEER challenge assays. Dead cells of *L. animalis* 506 failed to counteract the TEER decrease caused by either pathogen, suggesting that cell-associated factors of the strain are ineffective by themselves and that the protective effect of *L. animalis* 506 requires live cells.

Moreover, the effect of live *L. animalis* 506 demonstrated a dose-dependent response, as shown for the *S*. Heidelberg challenge model in Supplementary Figure S1. At 1 × 10^6^ CFU/transwell or lower, *L. animalis* 506 failed to show a protective effect on the cells.

Finally, *L. animalis* 506 also reduced TEER decrease and FD20 translocation across Caco-2 cell monolayers challenged with ruminant isolates of *S*. Newport and *S*. Dublin (Supplementary Figure S2).

### 3.2. L. animalis 506 and Other Probiotic Lactobacillus *spp.* Strains Regulate the Pathogen-Induced Intestinal Epithelial Inflammatory Responses

We next sought to examine the potential of the six *Lactobacillus* spp. strains to regulate the inflammatory response of the host intestinal epithelium to enteric pathogens. We, therefore, assessed the release of the pro-inflammatory chemoattractant cytokine IL-8 from enterocyte monolayers following exposure to the probiotic and pathogenic (*S*. Heidelberg and ETEC) strains. In a preliminary experiment using Caco-2 cells, the ETEC strain induced a minimal increase in IL-8 release. We therefore performed this set of experiments using HT29 cells, which are known to be more responsive than Caco-2 cells to inflammatory stimuli such as LPS [32]. Based on preliminary studies, the *Lactobacillus* spp. strains were administered at 1 × 10^8^ CFU/well, whereas *S*. Heidelberg and ETEC were administered at approximately 5 × 10^5^ and 1 × 10^5^ CFU/transwell, respectively, corresponding to the highest dosages at which the pathogenic strains did not cause visible signs of toxicity to the HT29 cells or made the cells detach from the wells. 

**Figure 2:**
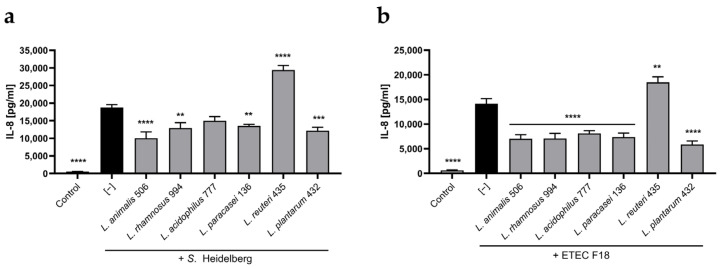
*L.animalis* 506 and other probiotic strains regulate pathogen-induced intestinal epithelial inflammatory responses. HT29 cell monolayers were incubated or not with probiotic *Lactobacillus* sp. strains for two hours, after which *S*. Heidelberg (**a**) or ETEC F18 (**b**) were added, and the co-cultures were incubated for an additional three hours. The supernatants were then aspirated, the cell monolayers were washed, and fresh media with gentamycin was added, followed by an additional 20 h of incubation. The supernatants were collected, and their IL-8 levels were quantified using ELISA. Data are expressed as means + SEM (n = 8). **** (*p* < 0.0001), *** (*p* < 0.001), and ** (*p* < 0.01) indicate significant differences from the respective “pathogen only” groups.

By themselves, the probiotic strains did not elicit an increased IL-8 release (Supplementary Figure S3). In contrast, both the *S*. Heidelberg and ETEC strains elicited an increase in IL-8 release, demonstrating their pro-inflammatory capabilities (Figure 2). Pre-incubation of the HT29 cell monolayers with *L. animalis* 506, *L. rhamnosus* 994, *L. paracasei* 136, and *L. plantarum* 432 significantly counteracted both *S*. Heidelberg and ETEC-induced IL-8 release, while *L. acidophilus* 777 only significantly counteracted ETEC-induced IL-8 release. In contrast, *L. reuteri* 435 failed to ameliorate the HT29 pro-inflammatory response to either pathogen.

Dead cells of *L. animalis* 506 failed to reduce the *S*. Heidelberg- or ETEC-induced IL-8 release from the HT29 cells, suggesting that the immune-regulatory effect of *L. animalis* 506 requires live bacterial cells (Supplementary Figure S4).

### 3.3. L. animalis 506 Counteracts the Damaging Effect of C. perfringens on Gut Barrier Integrity

**Figure 3:**
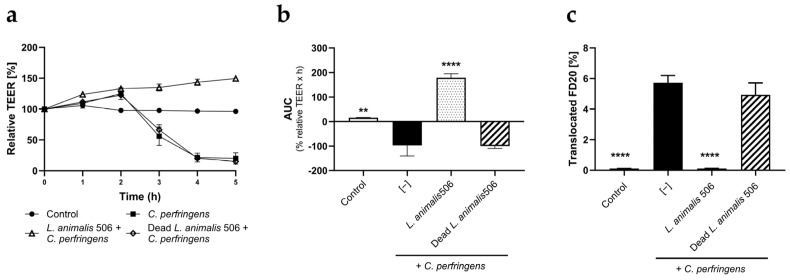
*L. animalis* 506 counteracts the damaging effect of *C. perfringens* on gut barrier integrity. Caco-2 cell monolayers were exposed on the apical side to *C. perfringens* in the presence or absence of *L. animalis* 506 and in the presence of FD20. TEER was measured for a total of 5 h, after which the amount of FD20 translocated to the basolateral compartment was quantified. Data are expressed as means of relative TEER (**a**), normalized AUC (**b**), and translocated FD20 (**c**) + SD (n = 3). **** (*p* < 0.0001) and ** (*p* < 0.01) indicate significant differences from the “*C. perfringens* only” group.

We next investigated whether *L. animalis* 506 can also attenuate the increased intestinal permeability caused by *C. perfringens*. Based on a preliminary dose–response study (Supplementary Figure S5), *C. perfringens* was administered at 2 × 10^7^ CFU/transwell. *C. perfringens* induced a rapid dose-dependent TEER decrease, with an approximately 80% decrease observed 4 h post-administration. In parallel, *C. perfringens* caused a significant increase in translocated FD20 quantified at the end of the 5 h experiment. Live *L. animalis* 506 counteracted the *C. perfringens*-induced TEER decrease and decreased FD20 translocation, whereas dead *L. animalis* 506 failed to show any effect (Figure 3a,b).

### 3.4. L. animalis 506 Ameliorates the Damaging Effect of Deoxynivalenol on Gut Barrier Integrity

We next investigated whether *L. animalis* 506 also confers in vitro protection of gut barrier integrity against the mycotoxin DON, a frequent contaminant of cereal-based food and animal feed [12]. DON impairs epithelial cell barrier integrity by inhibiting protein synthesis and directly affecting tight junctional proteins [33]. As rationalized by Akbari et al. [30], concomitant apical and basolateral exposure of epithelial cell monolayers to DON mimics the in vivo situation since DON is quickly absorbed in the upper parts of the small intestine while also likely being secreted to the intestinal lumen through the activity of ABC membrane transporters located on the apical surface of enterocytes [34,35,36]. Thus, simultaneous apical and basolateral exposure to DON was used in the following experiments. A dose-dependent damaging effect was observed with 50 µM DON, causing a 65% drop in TEER after 20 h, whereas only a minimal TEER decrease was observed at the lower tested dose of 25 µM (Supplementary Figure S6). Therefore, 50 µM was chosen for further experimentation. 

**Figure 4:**
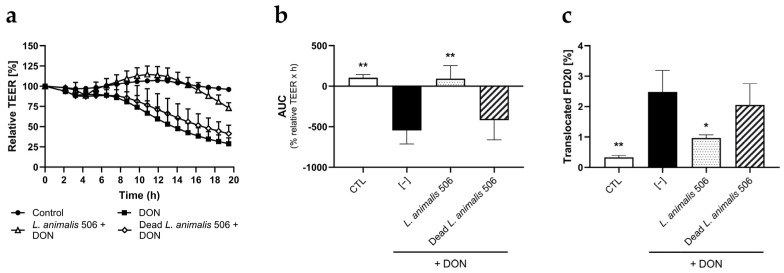
*L. animalis* 506 ameliorates the damaging effect of deoxynivalenol (DON) on gut barrier integrity. Caco-2 cell monolayers were exposed on the apical side and basolateral side to DON (50 µM) in the presence or absence of live or dead *L. animalis* 506 and in the presence of FD20, both on the apical side. TEER was measured for a total of 20 h, after which the amount of FD20 translocated to the basolateral compartment was quantified. Data are expressed as means of relative TEER (**a**), normalized AUC (**b**), and translocated FD20 (**c**) + SD (n = 3). ** (*p* < 0.01); * (*p* < 0.05) indicate significant differences from the “DON only” group.

As shown in Figure 4a,b, live *L. animalis* 506 significantly ameliorated the DON-induced TEER decrease, whereas dead *L. animalis* 506 conferred only a negligible protective effect. Correspondingly, live—but not dead—*L. animalis* 506 significantly reduced DON-induced FD20 translocation assessed after 20 h of mycotoxin/probiotic co-exposure (Figure 4b). Moreover, in line with the results from the TEER assays with enteric pathogen challenges, *L. animalis* 506 counteracted DON-induced TEER decrease to a greater extent than other *Lactobacillus* spp. strains (Supplementary Figure S7).

### 3.5. L. animalis 506 Mitigates the Effects of DON on Distribution Patterns of Tight Junction Proteins in Caco-2 Cells

Exposure of Caco-2 cell monolayers to DON has previously been shown to affect the cellular localization of TJ proteins, including occludin and claudins, as well as TJ scaffolding proteins such as ZO-1, which link the transmembrane TJ proteins to cytosolic actin filaments and other proteins [1]. We, therefore, assessed whether *L. animalis* 506 could counteract these DON-induced events, focusing here on occludin and ZO-1. 

**Figure 5:**
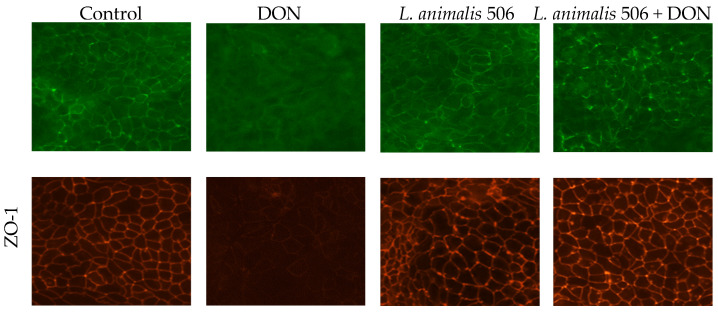
*L. animalis* 506 mitigates the effects of DON on the distribution patterns of tight junction proteins in Caco-2 cells. Caco-2 cell monolayers were exposed on the apical side and basolateral side to DON (50 µM) in the presence or absence of *L. animalis* 506. After 14 h, the cells were stained with occludin (green) and ZO-1 (red).

Caco-2 cell monolayers were incubated with or without *L. animalis* 506 and DON (50 µM) for 14 h, corresponding to a DON-induced TEER decrease of approximately 50%, followed by immunofluorescent staining. In the untreated Caco-2 cells or cells incubated only with *L. animalis* 506, we observed a clear peripheral localization of occludin and ZO-1 at the cell membrane (Figure 5). In contrast, exposure of the cell monolayers to DON caused a marked loss of both occludin and ZO-1 from the cell membranes. In the cells exposed to both *L. animalis* 506 and DON, the peripheral localization of occludin and ZO-1 was increased compared to DON-exposed cells. 

## 4. Discussion

In addition to its role in nutrient absorption, the intestinal epithelial barrier constitutes a major defense mechanism against invasion of luminal microorganisms or dietary antigens. An impairment of the intestinal barrier may trigger intestinal inflammation and possibly chronic pathological conditions [2,3]. Ingestion of enteric pathogenic bacterial species or mycotoxins of the trichothecene family are well-known factors that disrupt gut barrier integrity and function [6,7,20,37]. They are a particular concern for the health of livestock animals, which often face physical and/or environmental stressful conditions, e.g., weaning, transportation, feed and water deprivation, or heat stress, which may negatively impact their immune responses, rendering them more susceptible to infections [4,38,39]. Therefore, dietary supplements capable of supporting gut barrier integrity during stressful conditions are warranted in the field of animal health and nutrition.

*L. animalis* 506 is a probiotic strain used in commercial products to support the health and performance of beef and dairy cattle [24,25,26]. However, not much is known about the mechanisms underlying its proposed in vivo benefits. In this study, we used intestinal epithelial cell line-based in vitro assays to characterize the potential beneficial effects of *L. animalis* 506. We chose to focus on intestinal epithelial cells since probiotics are likely to interact directly with them in the small intestine and since epithelial cells are important regulators of inflammation, e.g., through the release of cytokines and chemokines, which in turn can impact local immune cells [40]. 

We first compared the ability of *L. animalis* 506 and five additional probiotic *Lactobacillus* spp. strains to support gut barrier integrity upon exposure to *S*. Heidelberg and ETEC, respectively. *S*. Heidelberg is among the most frequent *Salmonella* serovars reported for livestock in North America and has been associated with severe diarrheal illness in dairy calves [41]. On the other hand, ETEC is a primary cause of post-weaning diarrhea in nursery pigs and diarrhea in calves [42,43]. In line with similar in vitro studies [20,28], we found that most of the *Lactobacillus* spp. strains were able to counteract the TEER decrease and apical-to-basolateral translocation of FD20 across Caco-2 cell monolayers caused by the two pathogens. However, *L. animalis* 506 exhibited the most pronounced protective effect of all the tested strains, thus emphasizing its potential to confer beneficial effects. The strain furthermore supported gut barrier integrity in our in vitro model upon challenge with ruminant isolates of *S*. Newport and *S*. Dublin, two other serovars causing salmonellosis in cattle [6]. Notably, feed supplementation with *L. animalis* 506 (in combination with a *Propionibacterium freudenreichii* strain) has been shown to reduce the burden of *Salmonella* enterica in the lymph nodes of feedlot cattle, likely reflecting a reduced amount of *Salmonella* translocating across the intestinal mucosal barrier [25]. In another trial, administration of the same combination of *L. animalis* 506 and *P. freudenreichii* reduced diarrhea and other clinical manifestations in *Salmonella*-challenged beef calves [26]. Strengthening of the intestinal barrier, as demonstrated here in vitro, upon a *Salmonella* challenge may be one of the modes of action underlying the observed in vivo beneficial effects of *L. animalis* 506.

We next compared the capacity of the six *Lactobacillus* spp. strains to regulate inflammatory responses in HT29 cell monolayers exposed to the *S*. Heidelberg or ETEC strain, respectively. Specifically, we assessed the secretion of the pro-inflammatory chemokine IL-8 following exposure to the probiotics and/or the pathogens. IL-8 plays an important role in the recruitment and activation of neutrophils and thus may increase local inflammatory responses [40]. While the inflammatory response induced by the intestinal epithelium is an important host defense mechanism for controlling microbial infections, excessive and prolonged neutrophil infiltration may cause tissue damage and intestinal barrier dysfunction [44]. Notably, by themselves, none of the probiotic strains tested in this study elicited IL-8 release from the HT29 cells. However, in line with similar in vitro studies [20,45], we found that most of the probiotic strains ameliorated the increased IL-8 caused by the exposure of the cells to *S*. Heidelberg or ETEC. *L. animalis* 506 was among the strains exhibiting the most pronounced immunoregulatory effect. Thus, *L. animalis* 506 may be a good candidate for regulating IL-8 secretion from the intestinal epithelium to levels allowing for effective immune responses against invading enteric pathogens while not instigating excessive inflammation that could compromise the health and performance of humans and animals when a strengthened immune response is not needed. 

We also assessed the protective effect of *L. animalis* 506 against intestinal barrier damage caused by *Clostridium perfringens*. *C. perfringens* isolates are classified into types A-E based on their production of four major toxins (α, β, ε, and ι), of which type A only produces α-toxin [46]. Several additional toxins have been described in some *C. perfringens* isolates, of which *C. perfringens* enterotoxin (CPE) and δ-toxin are known to specially impair the intestinal epithelial barrier [47,48]. For our experiments, we chose a strain of *C. perfringens* type A, which is a common cause of necrotic enteritis in humans and livestock [7,8,49]. We found that *L. animalis* 506 was able to counteract the *C. perfringens*-induced profound and rapid TEER decrease and apical-to-basolateral FD20 translocation across the Caco-2 cell monolayers. Other research groups have demonstrated that probiotic bacterial strains can inhibit the growth of *C. perfringens* type A and its α-toxin production and cytotoxicity [50,51]. However, to our knowledge, our study is the first to demonstrate the ability of a probiotic strain to alleviate *C. perfringens*-induced impaired gut integrity in vitro. Interestingly, the *C. perfringens* type A used in this study produces neither CPE nor δ-toxin (NCBI whole-genome reference sequence: NC_008261.1), suggesting the capability of other *C. perfringens* toxins to impair intestinal epithelial integrity as well. 

Notably, we found that dead *L. animalis* 506 failed to impact the increased barrier permeability or the release of IL-8 from the epithelial cells upon challenge with the tested enteric pathogens, suggesting that cell-associated factors are insufficient to confer protective effects by *L. animalis* 506. Lactic acid bacteria, such as *Lactobacillus* spp. strains, may secrete a variety of antimicrobial compounds, such as short-chain fatty acids, lactic acid (causing pH reduction), or bacteriocins, which inhibit the growth of enteric pathogens [52,53,54]. They may also aggregate with the pathogens or compete with them for nutrients and binding sites on the intestinal epithelial cells by virtue of various surface proteins, such as pili [55,56,57]. Further studies are warranted to examine whether the protective effects of *L. animalis* 506 are attributed to specific secreted compounds and/or the interactions of the bacterial cells themselves with the pathogens or with the intestinal epithelial cells.

An objective of the present work was to investigate whether *L. animalis* 506 also confers protection to the intestinal epithelial barrier in vitro against the mycotoxin DON. Upon ingestion, DON may impair the integrity of the intestinal barrier, its first site of exposure. DON-induced increased epithelial permeability to toxins, pathogenic bacteria, and viruses then leads to gastrointestinal inflammation [16]. We reported that *L. animalis* 506 ameliorated the TEER decrease and increased apical-to-basolateral FD20 translocation across the Caco-2 cell monolayers caused by concomitant apical and basolateral exposure to DON. In contrast, dead *L. animalis* 506 exhibited only a negligible protective effect, suggesting the involvement of specific secreted bacterial compounds and/or the combined effects of such compounds and the bacterial cells themselves.

To further examine the underlying mechanism by which *L. animalis* 506 protects the epithelial barrier, we assessed the cellular distribution patterns of the TJ proteins occludin and ZO-1 in Caco-2 cell monolayers. In line with Akbari et al. [34], we observed a profound loss of both TJ proteins from the cell membranes upon exposure to DON, confirming that the DON-induced TEER decrease is accompanied by the reorganization of TJ proteins. However, the DON-induced occludin and ZO-1 redistribution was effectively counteracted by *L. animalis* 506.

Gu et al. [54] have previously shown that a heat-inactivated *Bacillus subtilis* strain could counteract DON-induced TEER decrease and disrupted ZO-1 membrane localization of IPEC-J2 porcine intestinal epithelial cell monolayers [58]. However, to our knowledge, our study is the first to demonstrate the protective effect of a non-spore-forming probiotic strain against DON in the in vitro model, as reported here. Moreover, Gu et al. [54] administered DON only in the apical compartment along with the probiotic strain, whereas in our model, DON was added both apically and basolaterally.

Previous studies have demonstrated the potential for probiotics to reduce the harmful gastrointestinal effects of DON in vivo and ex vivo. For instance, *Lactobacillus* spp. and *Eubacterium* spp. strains have been shown to alleviate morphological alterations of intestinal villi in broiler chickens administered DON-contaminated feed [59,60,61]. Moreover, a *Bacillus subtilis* strain proved to ameliorate impaired intestinal barriers, intestinal inflammation, and oxidative stress in piglets fed a DON-contaminated diet [62]. In addition, *Lactobacillus* spp. strains have been shown to decrease the histological alterations and intestinal permeability of pig jejunum explants exposed to DON ex vivo [63]. The proposed mode of action of the probiotic strains in these studies is adsorption or detoxification of DON, thereby reducing its negative effects in the intestine [59,60,64]. Interestingly, we found that basolateral DON administration resulted in almost the same level of TEER decrease as that of concurrent apical and basolateral DON administration, while apical DON administration only minimally impacted TEER levels, and *L. animalis* 506 also significantly counteracted basolateral DON-induced TEER decrease (Supplementary Figure S8). As *L. animalis* 506 was separated from DON by the Caco-2 monolayer, this rules out the adsorption of the mycotoxin as the primary underlying protective mode of action. Thus, our findings in this study elucidate an additional mechanism by which probiotics may confer protection from DON by directly strengthening the intestinal barrier integrity or reducing the sensitivity of the epithelial cells to DON cytotoxicity. 

Considering our focus on mimicking in vitro challenge conditions relevant to the gastrointestinal health of farm animals, in particular beef and dairy cattle, the use of human-derived cell lines is a limitation. A bovine intestinal epithelial cell line has been established in Japan and used to test probiotic strains [65]. Moreover, 2D and 3D bovine intestinal organoid systems have recently been developed [66,67]. Thus, it would be of great interest for future studies to evaluate the performance of *L. animalis* 506 or other probiotic strains or strain combinations in similar assays using either of these model systems. 

## 5. Conclusions

In summary, we have demonstrated the ability of the probiotic *L. animalis* 506 strain to support gut barrier integrity and regulate epithelial inflammatory responses in vitro upon exposure to enteric bacterial pathogens and the frequent food contaminant mycotoxin deoxynivalenol. These findings provide new insights into the potential benefits of *L. animalis* 506 as a feed additive for farm animals, but additional in vivo studies are warranted to evaluate such responses in ruminants.

## Data Availability

Data is contained within the article and Supplementary Material.

## Supplementary Materials

The following supporting information can be downloaded at https://www.mdpi.com/article/10.3390/ani14020269/s1, Figure S1: *L. animalis* 506 dose-dependently counteracts impaired gut barrier integrity caused by ruminant isolate of *Salmonella* Heidelberg; Figure S2: *L. animalis* 506 counteracts impaired gut barrier integrity caused by ruminant isolates of *Salmonella* Newport and *S*. Dublin; Figure S3: The probiotic *Lactobacillus* spp. strains do not elicit increased IL-8 release from HT29 cells; Figure S4: Dead *L. animalis* 506 fails to reduce *Salmonella* Heidelberg- or ETEC-induced IL-8 release from HT29 cells; Figure S5: *Clostridium perfringens* causes TEER decrease and increased FD20 translocation in a dose-dependent manner; Figure S6: Deoxynivalenol causes TEER decrease in a dose-dependent manner; Figure S7: *L. animalis* 506 excels among probiotic *Lactobacillus* spp. strains at mitigating DON-induced impaired gut barrier integrity; Figure S8: *L. animalis* 506 counteracts TEER decrease caused by DON administered only in the basolateral compartment.
