# Supplementary material for: Ligilactobacillus animalis 506 Protects the Intestinal Barrier from the Damaging Effects of Enteric Pathogens and Deoxynivalenol
Source: Animals (Basel). 2024 Jan 15;14(2):269. doi: 10.3390/ani14020269 (PMC10812616; doi:10.3390/ani14020269)
Supplement: Supplementary file 1 [file animals-14-00269-s001.zip › animals-2757357-supplementary.pdf]

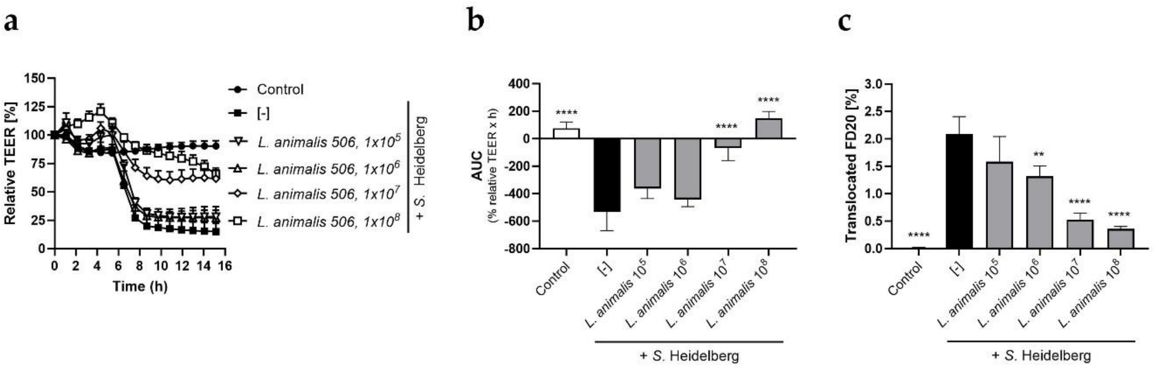

Figure S1. *L. L. animalis* 506 dose-dependently counteracts impaired gut barrier integrity caused by ruminant isolate of *Salmonella* Heidelberg. Caco-2 cell monolayers were exposed on the apical side to *S.* Newport at 2×10^7 CFU/transwell in the presence or absence of *L. animalis* 506 at 1×10^8, 1×10^7, 1×10^6 or 1×10^5 CFU/transwell and in the presence of FD20. TEER was measured for a total of 15 hours (**a**), after which the amount of FD20 translocated to the basolateral compartment was quantified (**c**). Data are expressed as means + SD for triplicate samples from one experiment. **** (p < 0.0001), ** (p < 0.01) indicate significant differences from the "*S.* Heidelberg only" group.

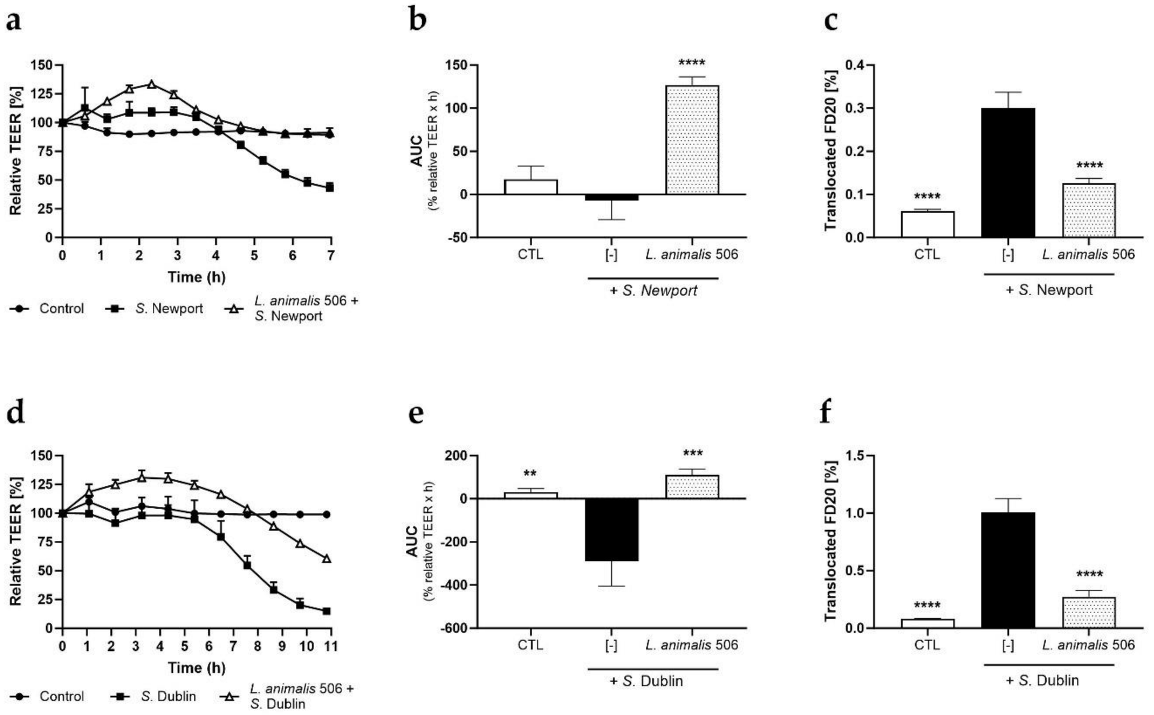

Figure S2. *L. animalis* 506 counteracts impaired gut barrier integrity caused by ruminant isolates of *Salmonella* Newport and *S*. Dublin. Caco-2 cell monolayers were exposed on the apical side to *S*. Newport (**a**- **c**) or *S*. Dublin (**d**- **f**) (approx. 2×10^7 CFU/transwell) in the presence or absence of *L. animalis* 506 (approx. 1×10^8 CFU/transwell) and in the presence of FD20. TEER was measured for a total of 7 (**a**) or 11 hours (**d**), after which the amount of FD20 translocated to the basolateral compartment was quantified (**c** and **f**). Data are expressed as means + SD for triplicate samples from one experiment. **** ($p < 0.0001$), *** ($p < 0.001$), ** ($p < 0.01$) indicate significant differences from the "pathogen only" groups.

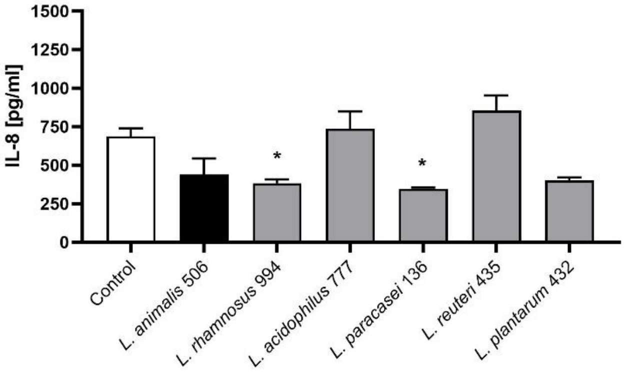

Figure S3. The probiotic *Lactobacillus* spp. strains do not elicit increased IL-8 release from HT29 cells. HT29 cell monolayers were incubated with *Lactobacillus* spp. strains for 5 hours, after which the supernatants were aspirated, the cell monolayers washed, and fresh media with gentamycin added followed by additional 20 hours of incubation. The supernatants were then collected, and their IL-8 levels quantified by ELISA. Data are expressed as means + SEM for one experiment with quadruplicate samples. * ($p < 0.05$) indicate significant differences from negative control group.

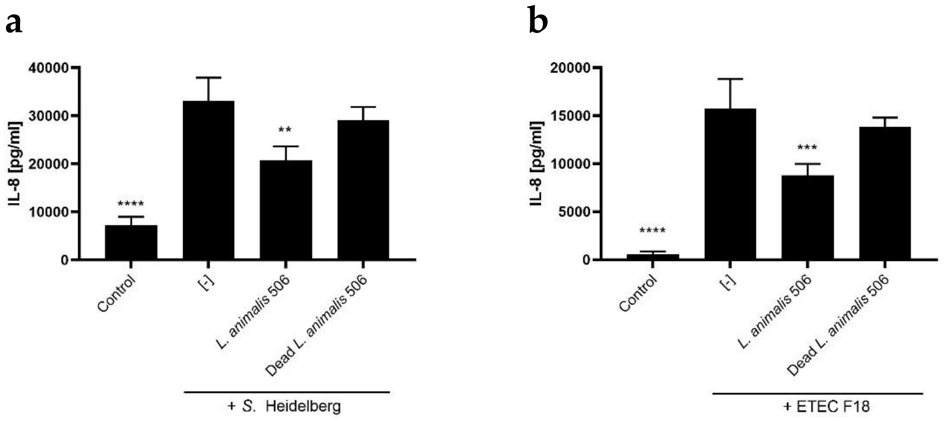

Figure S4. Dead *L. animalis 506* fails to reduce *Salmonella* Heidelberg- or ETEC-induced IL-8 release from HT29 cells. HT29 cell monolayers were incubated or not with live or dead *L. animalis* 506 for two hours, after which *S.* Heidelberg (**A**) or ETEC F18 (**B**) was added, and the co-cultures incubated for an additional three hours. The supernatants were then aspirated, the cell monolayers washed, and fresh media with gentamycin added followed by additional 20 hours of incubation. The supernatants were then collected, and their IL-8 levels quantified by ELISA. Data are expressed as means + SEM for one experiment tested in quadruplicates. **** ($p < 0.0001$), *** ($p < 0.001$), ** ($p < 0.01$) indicate significant differences from the respective "pathogen only" groups.

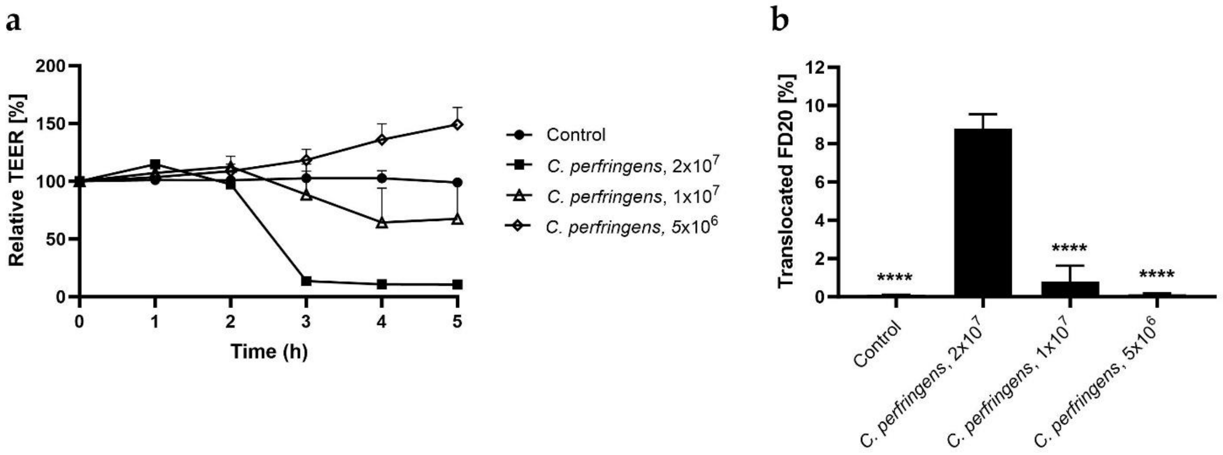

Figure S5. *C. perfringens* causes TEER decrease and increased FD20 translocation in a dose-dependent manner. Caco-2 cell monolayers were exposed on the apical side to *Clostridium perfringens* at 2×10^7, 1×10^7 or 5×10^6 CFU/transwell in the presence of FD20. Relative TEER was measured for a total of 5 hours (**a**), after which the amount of FD20 translocated to the basolateral compartment was quantified (**b**). Data are expressed as means + SD for triplicate samples from one experiment. **** ($p < 0.0001$) indicate significant differences from the "*C. perfringens*, 2×10^7 CFU/transwell" group.

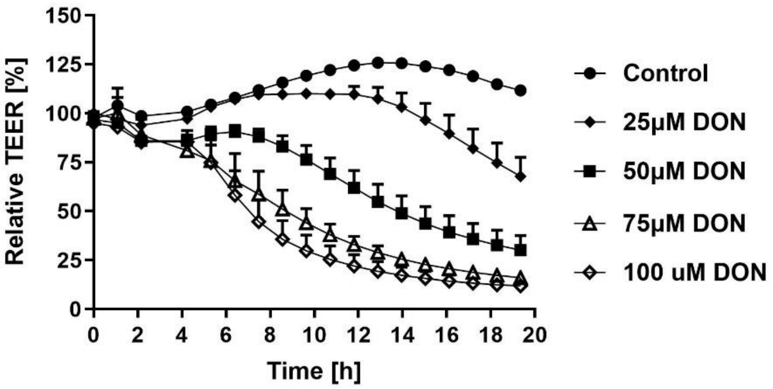

Figure S6. Deoxynivalenol (DON) causes TEER decrease in a dose-dependent manner. Caco-2 cell monolayers were exposed on the apical and basolateral side to DON at various dosages. Relative TEER was measured for a total of 20 hours. The data are expressed as means + SD for triplicate samples from one experiment.

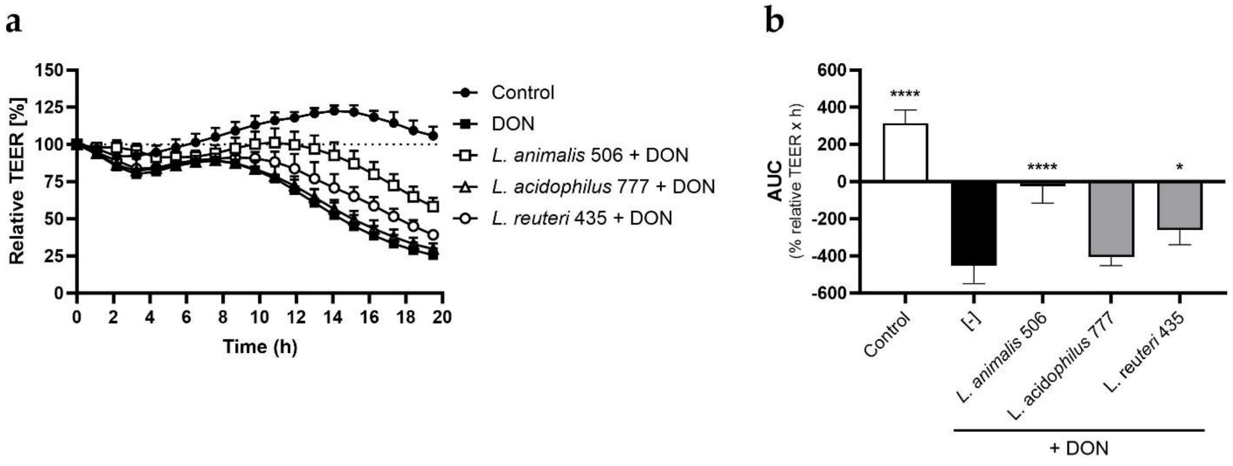

Figure S7. *L. animalis* 506 excels among probiotic *Lactobacillus* spp. strains at mitigating DON-induced impaired gut barrier integrity. Caco-2 cell monolayers were exposed on the apical and basolateral side to DON (50 μM) in the presence or absence of *L. animalis* 506, *L. acidophilus* 777 or *L. reuteri* 435. TEER was measured for a total of 20 hours. The data are expressed as means + SD for triplicate samples. **** ($p < 0.0001$), * ($p < 0.05$) indicate significant differences from the “DON only” group.

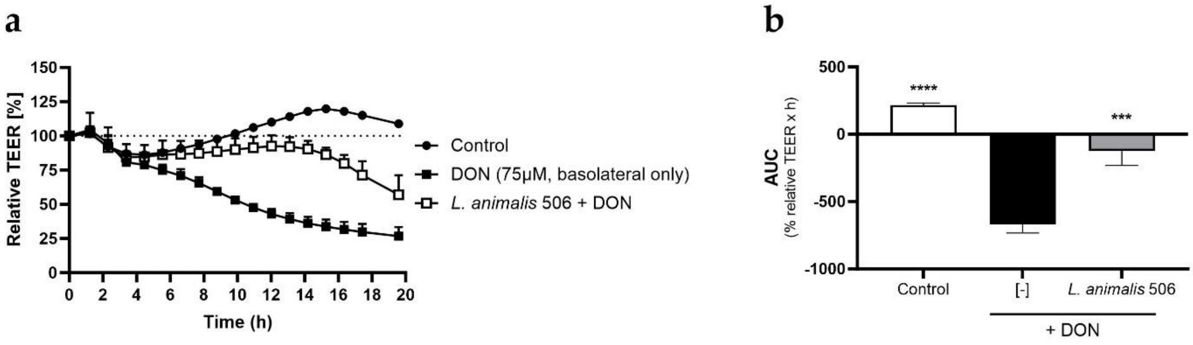

Figure S8. *L. animalis* 506 counteracts TEER decrease caused by DON administered only in the basolateral compartment. Caco-2 cell monolayers were exposed on the basolateral side to DON (75 µM) in the presence or absence of *L. animalis* 506. TEER was measured for a total of 20 hours. The data are expressed as means + SD for triplicate samples. **** ($p < 0.0001$), *** ($p < 0.001$) indicate significant differences from the "DON only" group
